# Editorial: Advances in tumor microenvironment, immunology and immunotherapy of breast cancer

**DOI:** 10.3389/fonc.2025.1664563

**Published:** 2025-09-04

**Authors:** Yonghong Zhang, Jihong Cui, Shaoquan Zheng, Kosuke Kawaguchi, Ying Lin

**Affiliations:** ^1^ Department of Breast Surgery, Breast Disease Center, The First Affiliated Hospital, Sun Yat-sen University, Guangzhou, China; ^2^ Institute of Precision Medicine, The First Affiliated Hospital, Sun Yat-sen University, Guangzhou, China; ^3^ Department of Breast Surgery, Kyoto University Hospital, Kyoto, Japan

**Keywords:** tumor microenvironment, immunology, immunotherapy, breast cancer, drug resistance

The tumor ecosystems consist of tumor cells embedded within a dynamic tumor microenvironment (TME) comprising immune cells, stromal components, and extracellular factors (1). In breast cancer, the TME exhibits remarkable heterogeneity across molecular subtypes, critically influencing tumor progression, treatment response, and clinical outcomes (2–4). These subtype-specific TME variations have transformed breast cancer management, particularly in immunotherapy and precision oncology approaches. This Research Topic, *Advances in Tumor Microenvironment, Immunology and Immunotherapy of Breast Cancer*, comprises five comprehensive reviews and five original research articles, which collectively explore the interplay between local TME dynamics and systemic mediators in breast cancer progression, therapeutic resistance mechanisms, and the discovery of novel prognostic biomarkers and therapeutic targets.

TME complexity significantly impacts disease characteristics and treatment efficacy. Xu et al. performed a comprehensive bibliometric analysis using Bibliometrix (R) and VOSviewer to map the 27-year evolution of triple-negative breast cancer (TNBC) clinical trials research globally. Their analysis highlights the groundbreaking advances in immunotherapy for TNBC treatment alongside the growing importance of antibody-drug conjugates (ADCs) in targeted therapy approaches. Wang et al. comprehensively reviewed the existing research on the resistance mechanisms of trastuzumab for human epidermal growth factor 2 (HER2+) breast cancer. One aspect in this review particularly emphasizes TME-mediated resistance pathways operating through both cell-autonomous and non-cell-autonomous mechanisms, including impaired antibody-dependent cellular cytotoxicity (ADCC), aberrant secretion of immunosuppressive cytokines, stromal remodeling, and altered immune cell infiltration.

Hypoxia is a significant feature of TME heterogeneity that arises from dysregulated tumor vasculature and heightened metabolic demand. Zhi et al. reviewed the latest research progress on the role of Hypoxia-inducible factor 1-alpha (HIF1α) in breast cancer progression and therapy resistance across molecular subtypes. Their work highlights HIF1α-mediated mechanisms including metabolic reprogramming, immune evasion, and treatment resistance, while also evaluating emerging therapeutic strategies targeting the HIF pathway in breast cancer patients.

Notably, rare tumor variants provide unique insights into TME plasticity that directly inform clinical decision-making. While high tumor-infiltrating lymphocytes (TILs) typically correlate with improved survival in TNBC, Jiao et al. reported a rare case of low-TIL TNBC with favorable prognosis, named Tall Cell Carcinoma with Reversed Polarity (TCCRP). Their mechanistic studies implicated that cancer-associated fibroblasts (CAFs) in the interstitium of the tumor contribute to immune evasion in this subtype. These findings suggest fibroblast subpopulation targeting could potentially overcome immunotherapy resistance in TCCRP patients, proposing a novel therapeutic strategy for this unique TNBC variant. Bai et al. reported a rare progesterone receptor (PR)-positive acinic cell carcinoma (AcCC) that exhibited distinct molecular features from conventional TNBC. The PR+/E-cadherin+ phenotype in this rare variant indicates unexpected endocrine responsiveness and targetable pathways, redefining treatment approaches for special breast cancer subtypes.

TME information can provide interpretable prognostic biomarkers. Tertiary lymphoid structures (TLS) serve as critical immunological hubs that orchestrate local antitumor responses and have emerged as robust prognostic biomarkers in cancer. In their comprehensive analysis of 95 treatment-naïve primary breast carcinoma specimens, Fang et al. systematically characterized TLS maturation stages and their associated immune cell composition. The study revealed that peritumoral TLS containing CD103+CD8+ tissue-resident memory T cells (Trm) and activated NK cells were associated with favorable clinical outcomes, with quantitative analyses demonstrating a progressive increase in these effector cell populations correlating with TLS maturation. These findings not only validate TLS as indicators of antitumor immune competence, but also reveal the specific immune cell populations, particularly CD8+ Trm cells and NK cells, that drive their protective effects in breast cancer. Beyond cellular components, non-cellular elements in the TME, like cytokines, chemokines, and other soluble factors, play crucial roles in shaping the tumor immune landscape, thereby significantly influencing anti-tumor immunity and treatment responses. Zheng et al. advanced this understanding by developing TSPRS (TGF-β pathway-related prognostic signature), a novel machine learning model that evaluates TGF-β activity-associated prognostic signatures. Their work demonstrated significant correlations between TSPRS scores, TME characteristics, and immunotherapy response.

Systemic modulators can profoundly influence cancer immunity beyond the local tumor microenvironment. For systemic physiological modulation, Koivula et al. conducted a clinical trial investigating the immunomodulatory effects of acute exercise in newly diagnosed breast cancer patients. Their results revealed that just 30 minutes of physical activity prior to treatment could significantly alter circulating immune cell populations, including increased CD8+ T cell and NK cell proportions alongside decreased myeloid-derived suppressor cells (MDSCs). Notably, these exercise-induced changes showed correlation with disease status, suggesting potential applications for prescriptive exercise regimens in cancer therapy. For perioperative interventions, Kadantseva et al. conducted a comparative analysis of inhalation anesthesia (IA) and total intravenous anesthesia (TIVA) in a cohort of 98 breast cancer patients, while no significant differences were observed in the postoperative neutrophil-to-lymphocyte ratio (NLR). However, the IA group exhibited measurable suppression of humoral immunity, which could theoretically impair tumor-specific antibody production and compromise the antitumor activity of immune cells. These findings highlight the underappreciated role of perioperative interventions on sculpting the immunological milieu and determining long-term clinical outcomes in breast cancer management. For autoimmunity-cancer interplay, Qi et al.’s documentation of breast cancer-ulcerative colitis comorbidity provides clinical validation for the bidirectional autoimmunity-cancer interplay, where chronic inflammation may promote carcinogenesis while cancer therapies can trigger autoimmune responses. Collectively, these studies underscore how physiological, pharmacological, and pathological systemic factors collectively shape anticancer immunity and clinical outcomes.

In conclusion, the TME orchestrates breast cancer progression through complex cellular and molecular networks. This Research Topic provides multidimensional insights into TME heterogeneity and delivers new targets and strategies for personalized therapy. The studies featured in this Research Topic collectively advance our understanding of TME immunology, highlighting promising therapeutic targets, resistance mechanisms and novel prognostic tools that may guide clinical decision-making. However, the extraordinary complexity of the TME demands continued investigation using increasingly sophisticated methodologies, including single-cell technologies, spatial transcriptomics, and artificial intelligence approaches. We hope this Research Topic will serve as both a valuable resource and an inspiration for future research aimed at translating TME insights into improved patient outcomes.
